# Improving Transition to Employment for Youth With Physical Disabilities: Protocol for a Peer Electronic Mentoring Intervention

**DOI:** 10.2196/resprot.8034

**Published:** 2017-11-16

**Authors:** Sally Lindsay, Jennifer Stinson, Mary Stergiou-Kita, Joanne Leck

**Affiliations:** ^1^ Bloorview Research Institute Holland Bloorview Kids Rehabilitation Hospital Toronto, ON Canada; ^2^ Department of Occupational Science & Occupational Therapy University of Toronto Toronto, ON Canada; ^3^ Lawrence Bloomberg Faculty of Nursing and Hospital for Sick Children Toronto, ON Canada; ^4^ Telfer School of Management University of Ottawa Ottawa, ON Canada

**Keywords:** social support, mentor, social inclusion, youth, disability, rehabilitation, occupational therapy

## Abstract

**Background:**

Although youth with disabilities have much to gain from employment readiness programs, they are often excluded from or have limited access to vocational programs. One encouraging approach to address gaps in vocational programming is through peer electronic mentoring (e-mentoring), which may facilitate a smoother transition to adulthood by offering support to enhance coping skills. Despite the increase in online communities, little is known about their impact on vocational mentoring for youth with physical disabilities and their parents.

**Objective:**

The aim of this paper is to develop, implement, and assess the feasibility of an online peer mentor employment readiness intervention for youth with physical disabilities and their parents to improve their self-determination, career maturity, and social support compared to controls.

**Methods:**

A mixed-methods feasibility randomized controlled trial (RCT) design will be conducted to develop and assess the feasibility, acceptability, and initial efficacy of the “Empowering Youth Towards Employment” intervention. Youth (aged 15 to 25) with physical disabilities and their parents will be randomly assigned to a control or experimental group (4-week, interactive intervention, moderated by peer mentors).

**Results:**

Data collection is in progress. Planned analyses include pre-post measures to determine the impact of the intervention on self-determination, career maturity, and social support. A qualitative thematic analysis of the discussion forums will complement the surveys to better understand why certain outcomes may have occurred.

**Conclusions:**

Our intervention includes evidence-informed content and was co-created by a multi-disciplinary group of researchers and knowledge users. It has the potential for widespread implications as a cost-effective resource to supplement educational and vocational programming for youth with disabilities.

**Trial Registration:**

Clinicaltrials.gov NCT02522507; https://clinicaltrials.gov/ct2/show/NCT02522507 (Archived by WebCite at http://www.webcitation.org/6uD58Pvjc)

## Introduction

### Background

Although cultivating an appropriate labor supply is critical for economic growth, Canada currently faces a labor shortage [1-3]. One response to enhancing the labor force is to include under-utilized groups, such as people with disabilities, who are critical to a successful economy. Despite the strong business case for hiring people with disabilities, youth with disabilities have consistently low employment rates (eg, half or less) compared to youth without disabilities [3-6]. Although youth with disabilities have much to gain from employment readiness programs, they are often excluded from or have difficulty accessing high school and community vocational programs [4-6].

One approach to address gaps in vocational programming for youth with disabilities is through peer mentoring, which can facilitate a smoother transition to adulthood by offering informational, practical, and emotional assistance to enhance coping skills [7-11]. Peer mentoring interventions for youth without disabilities have been shown to be safe, feasible, and a cost-effective alternative to traditional vocational services [12-14]. Research on mentoring for youth without disabilities has beneficial impacts on job training, educational attainment, social skills, self-esteem, and work ethic [11,15,16]. One main challenge in implementing mentoring programs is finding peer mentors who are able to meet face-to-face. Thus, electronic mentoring (e-mentoring) can provide an excellent platform to address this hurdle by increasing the availability and accessibility of peer mentors [16]. In addition, despite the increase in online communities, little is known about their impact on vocational peer mentoring for youth with physical disabilities.

Focusing on adolescents and young adults is critical because disadvantages are compounded for those who start life with a disability [17,18]. Youth with disabilities represent a unique population that faces a challenging transition with respect to developmental tasks, social development, and role functioning [18]. Moreover, increased attention is being paid to “emerging adulthood”, which is a distinct developmental period between ages 18 to 25 years. This period is characterized by identity explorations, instability, self-focus, and development of executive functioning. Such traits are vital for job skills and independence [19]. Such development periods represent a critical window of opportunity to optimize and solidify positive behaviors and prevent impaired work productivity for youth with and without disabilities [7,17,19]. Job skill development is critical to employability and a successful transition to adulthood [17]. A recent systematic review of employment readiness programs for youth with physical disabilities revealed only 8 empirical studies. However, they showed that they have potential to improve self-confidence, self-awareness, goal setting, and knowledge of career options [20]. Common intervention components included experiential learning, mentorship, and family involvement [20]. This review revealed that there is a limited availability of such evidence-based programs for youth with physical disabilities in Canada. To date, we have not identified any vocational programs that involve e-mentoring that have been rigorously evaluated and published in the peer-reviewed literature.

#### Parental Support for Employment

Although there are many factors influencing employment such as individual (ie, self-care, self-efficacy, independence skills, etc) and socio-environmental factors (eg, accessible transportation, societal attitudes towards people with disabilities), in this study we focus on parental support [12,20,21]. Our previous research suggested this is an area that is worthy of further attention to enhance employability of youth with disabilities [20,21]. Parents are a vital source of support for young people (with and without disabilities) and play a key role in youth’s decision to obtain employment [21,22]. Although parents often provide a positive influence for youth without disabilities, this is often not the case for youth with disabilities who encounter overprotection or discouragement regarding employment [21]. Research consistently shows that parents raising a child with a disability often struggle with encouraging independent skills, especially with self-care and transportation, which are essential elements of work readiness [23-25]. Therefore, there is a critical need for interventions fostering positive parent expectations and promoting youth autonomy for those with disabilities. Specifically, researchers have noted that more parent-to-parent connections are needed to help youth with disabilities transition to adulthood and improve their competitiveness in the workforce [10,21,26]. Although some employment readiness interventions have a parental component, little is known about their effectiveness and few, if any studies in the peer-reviewed literature, have an e-mentor approach [20]. Our intervention actively involves parents and a separate parent-to-parent mentorship component to help empower them to encourage independence skills among youth with disabilities.

#### The Need for Peer Mentoring Among Youth With Disabilities

A method to help address gaps in vocational programming is through peer mentoring [27]. There is a strong empirical basis for using mentoring as an intervention for youth who may be disadvantaged in work and school, such as those with disabilities [27]. Evidence accrued from reviews on the impact of peer support programs among adults without disabilities, and youth with and without disabilities, shows that they are a cost-effective way to augment vocational and educational services and promote positive behaviors, improve self-efficacy, quality of life, and employment [7,27-31]. A meta-analysis of key components of peer mentor interventions of youth without disabilities included trained mentors, monitored implementation, structured activities, and parental involvement [27]. Peer mentors can offer tangible, informational, and emotional support and companionship for youth (with disabilities) and parents [22]. However, little is known about the effectiveness of peer mentorship for youth with disabilities. Implementing a peer mentor intervention for youth with disabilities is critical because they are an overlooked and vulnerable population with unique social and vocational needs. They experience periods of developmental, emotional and social changes, and major life transitions compared to other youth [17]. Further, youth with physical disabilities encounter different challenges than youth with chronic illnesses because their condition is often visible and they also encounter difficulties in mobility, speech, independence, coping, stigma, and social exclusion [32]. Peer mentors could help address some of these issues.

#### Online Peer Support

The Internet is a medium for interaction that can influence learning and behavior change [33-35]. Virtual communities are increasingly being used for learning, informational, and social support [33-38] for people with and without disabilities. Given that technology is already an important component of adolescents’ social networks where most youth seek information and communicate over the Internet, e-mentoring interventions have potential to benefit youth with disabilities [29-41]. E-mentoring (through a secure website) is a new approach to mentoring that can provide career-related and psychosocial support by addressing many of the challenges inherent in face-to-face mentoring such as providing unlimited access to mentors, greater flexibility in establishing and sustaining relationships, and improved accessibility by removing physical and geographical barriers [42]. Despite the increase in online communities, little is known about their use and impact for vocational mentoring for youth with physical disabilities.

#### Peer-Moderated Versus Un-Moderated Online Support Groups

A moderator refers to a person who facilitates and reviews postings of discussants, censors the material, and often helps participants to feel at ease [43,44]. A review of moderated support groups (both chat room and bulletin board format) for cancer found that peer support groups provided encouragement, empowerment, information, and a sense of cohesion [45]. Others argue that participation in electronic discussions is often minimal without a moderator because of the lack of collaboration and encouragement of active learning [46,47]. Moderators in online support groups for people with disabilities are often untrained volunteers or health professionals who stimulate discussions by posting questions or topics of interest to the group [43,44]. It remains unclear how trained peer-moderated (versus un-moderated) online communities can influence learning, specifically vocational skills among youth with disabilities. Thus, the aim of our study is to explore how a peer-moderated employment readiness intervention influences self-determination, career maturity, and social support.

### Theoretical Framework of Peer Mentoring and Social Support

We draw on LaGreca’s [48] model of social support to understand the role of peer mentoring in improving employment readiness skills among youth. Peer mentoring is a form of social support and is defined as “the provision of emotional, appraisal, and informational assistance by a created social network member who possesses experiential knowledge of a specific behavior or stressor and has similar characteristics as the target population” (page 321) [49]. A peer refers to someone who shares common characteristics such as age, gender, and disability status along with individual interests. Peers are important because they can offer someone to relate to and empathize with the individual in ways that a non-peer would be unable to (eg, a healthcare provider or parent) [42,50]. In LaGreca’s model, there are 4 forms of social support: tangible, informational, companionship and belong, and emotional. [48]. Tangible support involves practical assistance and encouraging persistence and optimism for resolving problems, affirmation of a peers’ feelings and behaviors, and reassurance that frustrations can be handled [48]. Informational support involves providing advice, suggestions, alternative actions, feedback, and factual information [48-50]. The third form involves companionship and belonging where the reciprocal nature of interaction with appropriate accommodations for a person’s disability. Finally, emotional support refers to expressions of caring, empathy, encouragement, and reassurance and is often linked with enhanced self-esteem [48-50]. These forms of support are based on experiential knowledge rather than formal training. Therefore, peer support interventions fit within a social support model and are consistent with mentoring models where career-related support (ie, tangible and informational) and psychosocial support (ie, companionship, belonging, and emotional support) are provided [51]. Peer support may operate as a mediating process during an intervention including social comparison (ie, comparison of stresses, coping strategies, support resources), social exchange (reciprocity of support), and social learning (ie, role modeling, exchange of experiential knowledge) [48-50]. Within the context of our intervention, we hypothesize that peer mentors (for both youth and parents) will act as positive role models helping to increase all forms of social support while enhancing self-efficacy and parental empowerment [48-50].

Here, we use a mixed-method design to develop, implement, and assess the feasibility of an online peer mentor employment readiness intervention for youth with physical disabilities and their parents to improve their self-efficacy, career maturity, and social support compared to controls. A mixed-method design (ie, embedded qualitative randomized controlled trial [RCT]) allows us to test the impact of the intervention as well as the content of the discussion forums. We draw on a theoretical framework to inform our understanding of the role of peer mentoring in improving employment readiness skills among youth.

## Methods

### Objectives

The primary objectives of this project are (1) to develop and assess the feasibility, acceptability, and initial efficacy (ie, pilot RCT) of an e-mentor employment readiness intervention for youth with physical disabilities and their parents for improving self-determination, career maturity, and social support compared to controls; (2) to document the role of mentors in the discussion forum; and (3) to explore what types of social support are provided within the discussion forums. This protocol describes a methodology designed to develop and evaluate an online employment readiness intervention for youth with disabilities.

### Design

Our design involves a feasibility RCT, embedded qualitative design [52] to assess the feasibility and initial efficacy of the e-mentoring employment readiness intervention. Mixed-method designs are commonly used when qualitative methods are embedded within a RCT [52]. This mixed-method, prospective, intention-to-treat RCT study involves an intervention group that receives the employment readiness modules and a peer e-mentor and a control group that receives the employment readiness modules only but can interact with others in their group (no e-mentor). Pre- and post-surveys (immediately following the completion of the intervention) will be conducted with both groups (ie, intervention and control). The qualitative component of the study involves analyzing the content of the discussion forums (described below).

The gold standard Medical Research Council Framework for the development and evaluation of RCTs guided our design [53]. We focus on the development and feasibility phases (to establish theoretical underpinnings and modeling to test the feasibility of key intervention components) [53]. The rationale, design, content, and length of our intervention is based on the following systematic reviews conducted by our team: (1) employment readiness interventions for youth with physical disabilities [20]; and (2) best practices of peer mentorship for improving school and work outcomes for disabled youth [54]. In addition, we conducted the following scoping reviews: (1) improving the inclusion of people with disabilities in the workforce [55]; and (2) mentoring practices for a diverse workforce [56]. Needs assessments of youth with disabilities and their parents regarding informational support for employment were also conducted [17,21].

### Sample and Recruitment

Participants (youth and parents) are recruited through invitation letters from a pediatric rehabilitation hospital, disability organizations, and community centers via referrals and advertisements. This method has been useful for obtaining reasonable response rates in previous studies on employment among youth with physical disabilities [17,21,52]. Inclusion criteria for youth participants involves the following: (1) able to read and write in English; and (2) youth with a physical disability—we draw on the World Health Organization’s International classification of functioning to inform our understanding of disability which is defined as impairment, activity limitation, participation restriction whereby a disability and functioning are shaped by interactions between health conditions, and contextual factors (ie, diagnoses commonly seen at our hospital such as cerebral palsy, muscular dystrophy, spinal cord injury, amputation, etc); (3) currently enrolled in or have completed a high school diploma in the applied or academic stream (to screen for cognitive impairment); (4) aged 15 to 25; (5) have access to a computer and Internet; and (6) have no paid work experience. The rationale for this age group and also for not having paid work experience is youth with disabilities often start their first employment experience later than youth without disabilities [21]. We recognize that the intervention may be somewhat time intensive for the younger ages who may still be in school. However, we intend to run the intervention during the summer break, so this should not be a concern. Exclusion criteria involve those who recently completed or currently participating in another employment readiness or peer support intervention.

Inclusion criteria for parents include (1) the parent of a youth meeting the above inclusion criteria; (2) can read and write in English; and (3) have access to a computer with Internet. A youth or parent can participate if their respective child or parent does not.

### Setting

For the purpose of this study, participants (youth and parents) access a separate password-protected area of the AbilityOnline website.

### Youth Employment Readiness Modules

The content and length of our intervention is evidence-based (ie, informed by 2 systematic reviews and 1 scoping review conducted by our team) [20,54-56]. The youth intervention (delivered by youth peer mentors) consists of 12 modules (3 per week over 4 weeks) and includes the following: (1) introduction and goal setting; (2) aspirations (self-awareness and self-assessment); (3) and expectations (self-awareness and self-assessment); (4) job searching techniques; (5) marketing yourself (resumes and presentation); (6) job interviews; (7) managing disability at work (self-care, disclosure, accommodations); (8) getting ready to work (transportation and essential life skills); (9) family role in supporting employment; (10) learning from professionals with disabilities; (11) social networking and community resources; and (12) referrals and next steps. Each module contains informative webpages and interactive materials (articles, videos) which can be viewed at their own pace.

### Parent Modules

The parent modules (which were co-created with parents of youth with disabilities) include (1) introductions, (2) life skills, (3) managing disability, (4) family role in supporting employment, (5) aspirations and expectations for work, (6) volunteerism, (7) finding a job or volunteer position, (8) social networking and community resources, (9) helping youth prepare for job interviews, (10) learning from professionals, (11) career pathways and transitions after high school, and (12) referrals and next steps.

### Intervention

The purpose of the intervention (for both youth and parents) is to provide meaningful support and access to evidence-based employment resources. The intervention, which was co-created with a knowledge user advisory group, consists of a 4-week, multi-component, interactive treatment of employment readiness modules, homework, and discussions led by trained peer mentors. The group-based intervention (10 participants per group with 4 groups, plus mentor) is hosted on the existing secure online peer networking AbilityOnline website designed for youth with disabilities and their parents.

Youth mentors present each of the topics to mentored participants in monitored interactions. They provide their own personal experiences and examples related to each topic and respond to all posts, offering informational, emotional, and social support. The discussion forum is available to the entire group and available only to participants and mentors in that particular group (ie, they cannot see participants’ discussions that are in another group). There is an option where youth can have private chats between members or with a mentor (ie, others cannot see what they say). Although we are not monitoring the content of private discussions for research purposes we log the number of private chats for each participant. Participants (youth and parents) decide when they want to log in and contribute at a time that is convenient for them. Mentors post their availability when they will be on in case participants (youth and parents) want to discuss something in real-time. There are also chat rooms that are in real-time if participants want to connect with others who are online at the same time. We instruct youth and parents to use pseudonyms and we remind them that all information shared within the forum should remain confidential and not disclosed to others.

### Parents’ Intervention Forum

The parent's intervention forum follows a similar design to the youth forum and includes a peer-led discussion forum (separate from youth) that contains relevant resources (for each of the topics mentioned earlier) and hosted through the AbilityOnline website. The parent’s forum consists of a 4-week, multi-component, interactive treatment of how to support their youth getting started with employment through modules and discussion. The group-based intervention (10 participants per group with 4 groups and a mentor) is hosted through AbilityOnline *.* A trained parent peer mentor emails each parent to determine module completion, posts their own personal experiences and reflections, responds to all posts, and offers informational, emotional, and social support.

### Control Groups

The control groups (for both parents and youth) have access to the modules only and do not receive peer mentorship. A researcher posts the discussion topic for the week but does not reply or encourage any follow-up discussion. Youth and parent participants are able to discuss topics and interact with others in their group but it is not facilitated by a mentor.

### Peer Mentor Training

All mentors (ie, 2 youth and 2 parents) are recruited through advertisements at a pediatric rehabilitation hospital, undergo rigorous screening (background checks and interviews to ensure appropriate fit and experience), and complete a Youth Peer Mentor Training Program [57] or Family Leader Training Program [58] prior to starting. Mentors (young adults with a disability with job experience or parents of disabled youth) are trained on how to use the AbilityOnline platform. Mentors introduce the topics in the same order and are trained to respond to participant’s comments in a similar manner—providing informational, appraisal, and emotional support. Prior to working with youth or parents, mentors practice their skills with fellow mentors whose recent experiences are similar to those of mentored participants (eg, training on active listening, perspective taking, confidentiality, maintaining boundaries, positive modeling, trust building through interactive training, and mentoring).

### Feasibility and Sample Size

To test the primary hypothesis that a peer-mentored employment readiness intervention will have better self-determination (Arc’s self-determination) [59], career maturity inventory [60], and parental support (multi-dimensional scale of perceived social support) [61] compared to youth in a non-mentored group, it is expected that a *t* test will be used. Following the guidelines of Cohen [62] and Hertzog [63] we estimated that with an alpha of .05, power of 80%, and a medium effect size (ie, 0.50), a sample size of 80 (40 in each group; experimental and control) is needed [62]. This sample is suitable for a feasibility pilot [62-64]. Data collection will take 18 to 24 months (recruited in 10 by 8 groups), including control and experimental over 4 weeks.

### Procedures and Randomization

Ethical approvals will be obtained from a pediatric rehabilitation hospital and a University Research Ethics boards prior to starting. Informed consent will be acquired from all participants prior to taking part. Once participants have consented, a blocked randomization method will be used. Using a block size of 10, participants are randomly assigned to the appropriate treatment condition as they enroll in the study until the block is completed. Then the following 10 participants are assigned to the next block [65,66]. Participants are blinded to each other, unaware of any manipulation. To avoid contamination, we asked participants not to share their password.

### Quantitative Data Collection

After randomization, a member of the research team asks each participant (parents and youth) to complete an online survey (pre-test) via Fluid Surveys (approximately 30 minutes to complete), stored on a secure site at a pediatric rehabilitation hospital. When complete, they are given password-protected access to the intervention through AbilityOnline. Following the intervention, participants complete a post-test survey containing the measures listed below. Demographic measures include: age, gender, type of disability, any assistive devices, education level, and access. Use and comfort level with computers and the Internet is collected at baseline to describe control and experimental groups and to assess whether they have similar characteristics.

Primary youth outcome measures include the following standardized measures, all of which have good internal consistency, construct related and criterion validity, test-retest reliability, and have been widely used for youth with disabilities [59-61]: (1) Career Maturity Inventory-Attitude Scale [60,67], a 25-item agree-disagree scale where responses form the basis for 5 subscales relating to career decision-making, including orientation, involvement, independence, compromise, and decisiveness; and (2) Arc’s Self-Determination Scale, a self-report measure that assesses self-determination for adolescents with disabilities [59] with subscales on autonomy, acting on the basis of preferences and abilities (post-school directions), goal setting, and task performance.

Measures used for both parents and youth include (1) the Multi-dimensional Scale of Perceived Social Support, a self-report measure assessing sources of social support [61]; (2) Family Empowerment Scale, a 34-item rating scale to measure empowerment in families with children who have a disability [68,69]; and (3) the Ragins and McFarlin Mentor Role Instrument [70,71] that assesses perceptions of mentoring relationships based on 5 mentoring roles in the career-related dimension and 6 mentoring roles in the psychosocial dimension and was developed based on Kram’s theory [51] of mentor roles. Secondary measures include online usage such as number of modules completed and usage patterns (ie, number of times logged in, length of time spent online, number and content of postings). These analytics will be built into the web-hosting Drupal platform [72].

## Results

Data collection for this study is in progress. The proposed analysis is outlined in further detail below.

### Quantitative Data Analyses

Quantitative data will be analyzed using SPSS, version 22. Rates of accrual, dropout, and compliance (ie, attendance and number of postings) will be calculated. Descriptive statistics will be used to provide an overview of sample characteristics using means and standard deviations for continuous factors and frequencies and proportions for categorical factors. We will use an intent-to-treat approach to our analysis. It is expected that *t* test and chi-squared analyses will be conducted to test intervention effects (comparing baseline primary outcome measures (time 1) and post-test (time 2) data. Separate analyses will be conducted for each outcome. To control for type I error rate, Holm’s sequential correction will be applied. Effect sizes for *t* tests and Cohen *d* will be reported [62]. A *P* value of less than .05 will be used as the criterion for statistical significance.

### Qualitative Data Analysis

Qualitative data analysis will be used to address objectives 2 and 3 and will consist of the following: (1) open-ended questions in post questionnaires including what participants liked most and least about each module and satisfaction with the intervention (benefits, challenges, and suggestions for improvement); and (2) transcripts of the intervention discussion forums. This data will be combined with the survey data to better understand why certain difference may have occurred.

Transcripts of all open-ended survey questions, discussion forums (for both the experimental and control groups), and open-ended questions on the survey will be entered into Nvivo, 10. The analysis will begin with at least 2 investigators independently reading all transcripts. Our research questions will guide the analysis of key themes emerging from the data. An open-coding content analysis will be used to understand the role of mentors (objective 2) and types of social support provided in the forums (objective 3) [73]. We will note key common meaning units (codes) around employment readiness, social support, role of peer mentors, aspirations and expectations of work environments, and co-creation of knowledge. A constant comparative approach will be used until consensus is reached among the research team on the final coding scheme. Several strategies will be used to ensure rigor and trustworthiness (transferability, dependability, conformability) of the qualitative findings including prolonged engagement, peer debriefing, and rich descriptive accounts with quotes reflective of the range of ideas expressed by participants [73,74].

### Combining Data

A mixed-method, embedded qualitative RCT design [52] will help us understand any discrepancies between expected and observed outcomes, provide insight into participant experiences and reasons for their preferences, explain how e-mentors influenced employment readiness skills (for youth) and empowerment (for parents), and what types of social support were provided. Our objectives combine quantitative and qualitative data and we will follow the guidelines of embedded qualitative RCT analysis [52,73-75]. Our findings will inform the feasibility and initial efficacy of an e-mentor employment readiness intervention for youth with physical disabilities. We hypothesize that participants in the intervention group will have significantly higher career maturity scores, self-determination, perceived social support, and family empowerment compared to controls.

## Discussion

### Principal Findings

This project is timely and significant: the United Nations Convention on Rights of Persons with Disabilities [76] stresses the need for people to have opportunities for freely chosen work and access to guidance programs and training. There is currently a lack of e-mentoring employment readiness interventions for youth with physical disabilities. No RCTs have been conducted on the feasibility and efficacy of online employment readiness programs for youth with physical disabilities. The goal is for this innovative approach to optimize vocational skills for youth with disabilities.

Our research addresses several important gaps in the literature. First, there is a lack of theory-driven, evidence-based employment readiness interventions for youth with physical disabilities. The few programs that do exist have not been rigorously evaluated, have small sample sizes, and lack random assignment and comparison groups [18,42]. Applying a theoretical framework could help standardize the essential ingredients of a job readiness program including peer mentors [7,37]. Second, most programs are inaccessible to youth. Third, although there are an increasing number of online peer support programs, we have not seen any that are evidence-based in the peer-reviewed literature that focus on employment readiness for youth with physical disabilities. Fourth, little is known about the role of peer-moderated online support versus un-moderated support [43].

### Conclusion

Although the intervention is being evaluated in the context of youth with physical disabilities, it has potential to be used across a range of other age groups and health conditions. Given that people with disabilities are an under-represented population within the labor market, the findings could help inform needed supports related to accessing employment for this diverse group. Our intervention, co-created by a multi-disciplinary group of researchers (community members, knowledge users, and policy advisors), can serve as a stepping-stone to greater accountability and standardization of support services for students with disabilities and has potential for widespread implications as a cost-effective resource to supplement educational and vocational programming for youth with disabilities.

## Abbreviations

RCT: randomized controlled trial
e-mentor: electronic mentor
e-mentoring: electronic mentoring
